# Cryoablation synergizes with anti-PD-1 immunotherapy induces an effective abscopal effect in murine model of cervical cancer

**DOI:** 10.1016/j.tranon.2024.102175

**Published:** 2024-11-02

**Authors:** Xiaoming Yang, Xiaoyan Gao, Chen Xu, Ting Ni, Yaru Sheng, Jing Wang, Xiao Sun, Jiangjing Yuan, Lin Zhang, Yudong Wang

**Affiliations:** a Department of Gynecologic Oncology, the International Peace Maternity and Child Health Hospital, School of Medicine, Shanghai Jiao Tong University, Shanghai 200030, China; b Shanghai Municipal Key Clinical Specialty of gynecologic oncology, Shanghai 200030, China; c Shanghai Key Laboratory of Embryo Original Diseases Affiliated to Shanghai Jiao Tong University School of Medicine, Shanghai 200030, China

**Keywords:** Cervical carcinoma, Cryoablation, Tumor immunotherapy, PD-1

## Abstract

**Background:**

Immune checkpoint inhibitors (ICIs), especially anti-PD-1/PD-L1 antibodies, have emerged as promising therapeutic options for cervical cancer. However, the efficacy of anti-PD-1 antibody monotherapy is limited. Cryoablation could elicit an anti-tumor immune response, thereby presenting itself as a potential approach to augment the response of ICIs. The aim of our study was to investigate the systemic immunological effects of cryoablation and the potential synergistic anti-tumor effects of cryoablation and anti-PD-1 antibody in cervical cancer.

**Methods:**

We established U14 murine bilateral subcutaneous cervical cancer model, wherein the primary tumors were treated with cryoablation. Flow cytometry, immunohistochemistry and RNA-seq were used to analyze the immune cell infiltration and immune-associated pathways in the secondary tumor.

**Results:**

Our study revealed that cryoablation reprogrammed the immune landscape, leading to an enhanced infiltration of CD8^+^ T cell in distant tumors. Cryoablation created a conducive environment for increasing the efficacy of anti-PD-1 immunotherapy. Cryoablation in combination with anti-PD-1 antibody inhibited distant tumors growth and improved mouse survival. Mechanistically, this combination therapy could augment the infiltration of CD8^+^ T cells, CD4^+^ T cells, dendritic cells and M1-like tumor-associated macrophages, enhance multiple aspects of antitumor immune response, and reduce immunosuppressive cells such as M2-like tumor-associated macrophages and myeloid-derived suppressor cells in distant tumors.

**Conclusions:**

Combination therapy with cryoablation and anti-PD-1 antibody induces an effective abscopal effect in murine model of cervical cancer and may be a novel therapeutic approach for patients with advanced/recurrent cervical cancer.

## Highlights

• Cryoablation triggered an inflammatory process in distant tumor, turned the TME from immunologically “cold” into “hot”.
• Cryoablation combined with anti-PD-1 immunotherapy synergistically enhances antitumor immunity.
• Our findings offer preclinical evidence supporting the therapeutic application of cryoablation in combination with anti-PD-1 antibody for cervical cancer patients.

## Graphical abstract

The combination of cryoablation and anti-PD-1 antibody can jointly improve antitumor immune responses by potentiating the antitumor effects and reversing the immunosuppressive nature of the cervical cancer TME.

**Figure:**
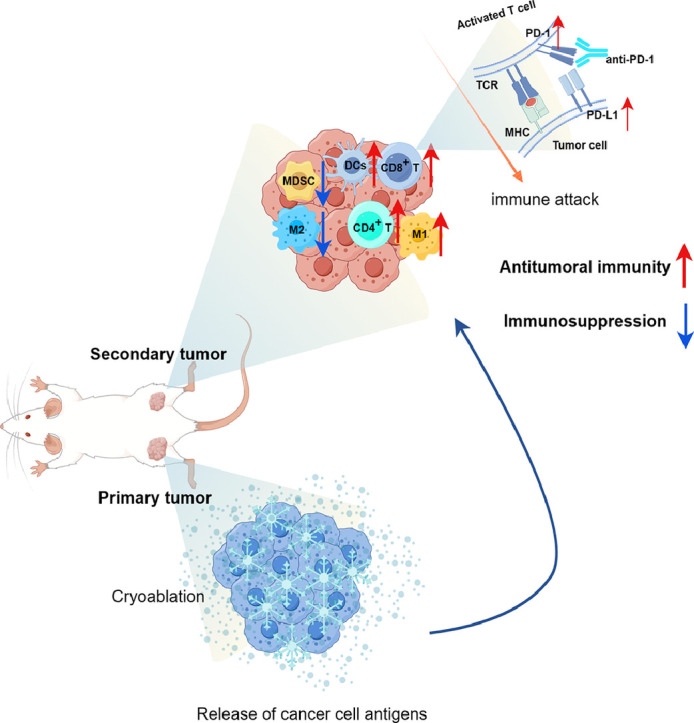

## Background

Cervical cancer ranks as the fourth most prevalent malignancy among women worldwide [1]. However, individuals with advanced or recurrent cervical cancer face limited effective treatment options, resulting in significantly lower 5-year survival rates (10%) compared to those diagnosed early (up to 90%) [[1], [2], [3], [4]]. Recurrence and metastasis of cervical cancer are major factors affecting patient prognosis.

Immune checkpoint inhibitors (ICIs), specifically programmed death 1 (PD-1)/programmed death ligand 1 (PD-L1) inhibitors, have been employed in the treatment of cervical cancer. However, the anti-PD-1 antibody monotherapy has demonstrated limited therapeutic efficacy, with an objective response rate (ORR) of 12.2% for the overall population and 14.6% for PD-L1 positive patients [5]. Consequently, there is an urgent need to enhance the therapeutic efficacy of ICIs and explore novel combination regimens for cervical cancer.

Cryoablation is an ablation technique that utilizes extreme low temperatures for tumor destruction. Owing to the rapid development of imaging techniques and cryoablation devices, cryoablation is widely used in the clinical treatment of various solid tumors, such as liver cancer [6], non-small cell lung cancer [7], prostate cancer [8] and breast cancer [9]. We propose that cryoablation holds significant potential in the comprehensive treatment of cervical cancer. For advanced and recurrent cervical cancer, cryoablation can achieve the goals of reducing lesion size, halting bleeding, and ameliorating symptoms. Moreover, cryoablation has the potential to induce abscopal effect, characterized by the reduction or disappearance of cancer foci in non-frozen regions [10]. Consequently, cryoablation may serve as a viable approach to enhance the response rates of ICIs.

Nevertheless, it is uncertain whether cryoablation can trigger anti-tumor immune responses in cervical cancer, and which immunotherapy regimens combined with cryoablation can generate the best tumor killing effect. As a clinically approved PD-1 antibody, Pembrolizumab was the first ICIs recommended by National Comprehensive Cancer Network (NCCN) guidelines for the treatment of cervical cancer and showed better antitumor activity [11]. Therefore, our initial conjecture revolves around the potential utilization of PD-1 antibodies in conjunction with cryoablation.

In this study, we describe an immunotherapeutic strategy for cervical cancer that reshapes tumor microenvironment (TME) to anti-tumor responsiveness through the synergistic utilization of cryoablation and anti-PD-1 antibody. The findings of this research provide preclinical evidence supporting the combination of cryoablation and anti-PD-1 immunotherapy for the treatment of advanced/recurrent cervical cancer.

## Methods and materials

### Tumor cell line

The U14 cell line is a mouse cervical undifferentiated squamous cell carcinoma cell line. This cell line has stable biological characteristics and can form tumors subcutaneously in inbred mice, which is widely used in tumor immunity research. For this study, U14 cells were cultured at 37 °C with 5% CO_2_ in DMEM high glucose culture medium containing 10% fetal bovine serum.

### Immunofluorescence staining

Immunofluorescence staining was used to detect CRT exposure. Briefly, U14 cells were frozen in refrigerators at −20 °C, −40 °C or −80 °C for 20 min and then put at 37 °C for 10min. The cells were then washed, fixed, blocked, and incubated with anti-Calreticulin antibody (27298–1-AP, Proteintech) at 4 °C overnight. Next, the cells were washed and stained with FITC-conjugated secondary antibody. Lastly, the cells were labeled with DAPI and observed under CLSM.

### Quantification of ATP release

U14 cells were frozen in refrigerators at −20 °C, −40 °C or −80 °C for 10 min or 20 min and then put at 37 °C for 10min. The supernatants were collected and centrifuged at 12,000 rpm at 4 °C for 3 min. The ATP levels were measured using ATP assay kit (Beyotime, China) according to the manufacturer's instructions. Luminescence was measured using BioTek Synergy^TM^ H1 microplate reader.

### Murine cervical cancer model and *in vivo* treatment

BALB/c mice (4–5 weeks old, female) were used in the current study, and these mice were bred in individual ventilated cages (IVC) under SPF conditions and given standardized diets with a maximum of five mice per cage in the Animal Science Centre at the Shanghai Jiao Tong University School of Medicine. The experimental procedures with animals were approved by the Ethics Committee of International Peace Maternity & Child Health Hospital, School of Medicine, Shanghai Jiaotong University. The bilateral murine subcutaneous cervical cancer model was established as for the *in vivo* anticancer efficacy. Detailed information on the construction of murine model is provided in supplementary information.

Cryoablation was carried out using a liquid nitrogen-based cryoablation device (SensCure Biotechnology, Ningbo, Zhejiang, China). We adopted a “rapidly freezing-slowly thawing-double cycle” cryoablation mode to achieve complete tumor destruction [12,13]. The approach to performing cryoablation and detailed information on experimental design can be found in supplementary information.

### Flow cytometry

Samples, including tumors and spleens were collected on day 12 for experiments. At the time of harvest, mice were euthanized using CO_2_. Fresh mouse tumor samples were dissociated into single cell suspensions by using Mouse Tumor Dissociation Kit (Cat# 130–097–730) obtained from Miltenyi Biotech, following the manufacturer's instructions. Mouse spleen single-cell suspensions were prepared by standard procedures. All single-cell suspensions were made and passed through a 70 µm cell strainer to ensure single-cell suspension. Subsequently, the single-cell suspensions were stained with fluorochrome-conjugated antibodies listed in Table S1. All samples were detected by FACS Calibur Flow cytometry (BD, USA) in our laboratory and the data were analyzed by FlowJo version 10.6.2 (www.flowjo.com). Flow cytometry gating strategy were showed in Fig. S3.

### Immunohistochemical analysis

Paraffin-embedded sections of secondary tumors from mice in each group were dewaxed with environmentally friendly dewaxing agent (Solarbio, China), hydrated with gradient alcohol, repaired in the EDTA antigen repair buffer (pH 9.0) under high pressure and high temperature for 90s. Then sections were hydrated with 3% hydrogen peroxide to quench endogenous peroxidase, followed by blocked in 10% of normal goat serum. The sections were incubated overnight at 4 °C overnight with primary antibodies, including PD-L1/CD274 Polyclonal Antibody (17952–1-AP, Proteintech), CD8a Monoclonal Antibody (14–0808–82, eBioscience) and Granzyme B Monoclonal Antibody (14–8822–82, eBioscience), followed by secondary antibody and visualized with diaminobenzidine (DAB). Detailed immunohistochemical staining scores for PD-L1, CD8, and Granzyme B can be found in supplementary information.

### RNA seq analysis

On day 12, the mice were sacrificed and the secondary tumor tissues were harvested and analyzed by RNA-Seq, which was performed using Illumina NovaSeq™ 6000 platform in OE Biotech, Inc., Shanghai, China. The gene set variation analysis (GSVA) was used to explore the differential activity of molecular pathways between different experimental groups by using the “GSVA” package in R software [14]. T cell cytotoxicity score, T cell co-stimulation score, and Th1 cells score were analyzed by using single-sample gene set enrichment analysis (ssGSEA). The detailed list of gene sets was shown in Table S2. Activated or repressive biological pathways were detected using gene set enrichment analysis (GSEA), FDR < 0.25, p-value <0.05, and |NES| > 1 were considered statistically significant. Gene Ontology (GO) gene sets were downloaded from MsigDB (http://www.broadinstitute.org/gsea/index.jsp). The infiltration of immune cells in murine tumors was evaluated using mMCP-counter algorithm [15].

### Statistical analysis

Statistical analysis was performed using SPSS 20.0 software (IBM SPSS 20.0, SPSS Inc.). Data are expressed as Mean ± SEM. Comparisons between two groups were performed using Student's *t*-test (two-tailed). Correlations were analyzed with Pearson correlation analysis. Survival data were analyzed using Kaplan-Meier survival analysis with the log-rank test. *P* value of <0.05 was considered to be significant. Fishers exact test was adopted to test the comparison of effective rate between two groups.

## Results

### Cervical cancer cryoablation creates suitable conditions for anti-PD-1 immunotherapy

**Fig. 1:**
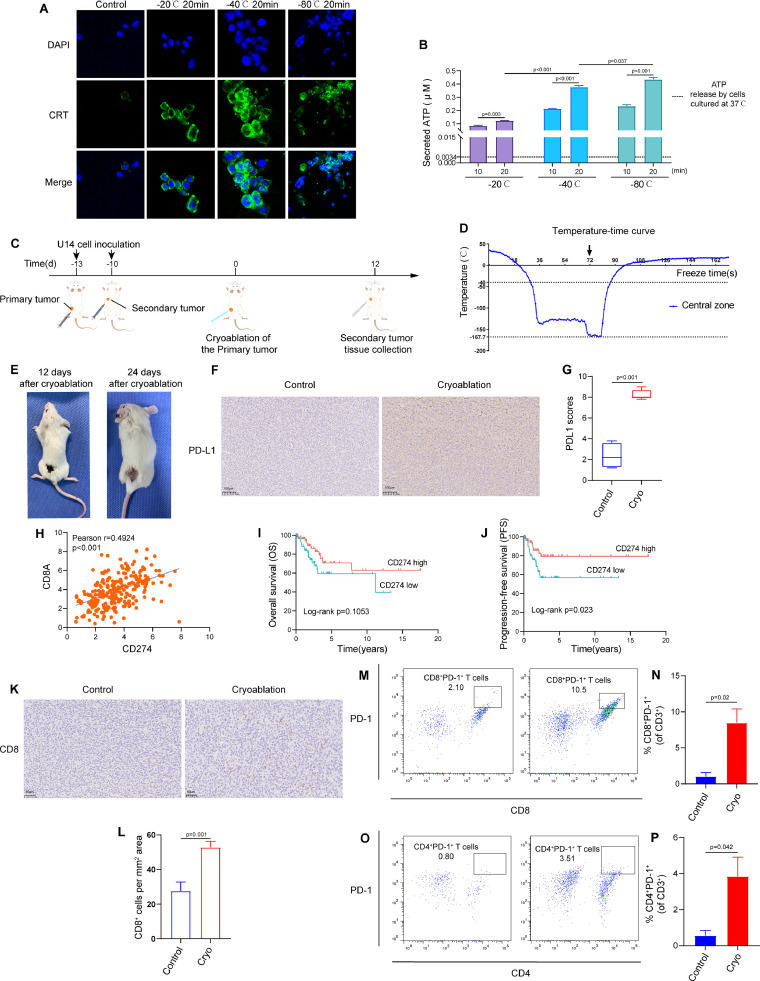
**Cervical cancer cryoablation creates suitable conditions for anti-PD-1 immunotherapy in distant tumors.** (**A)** Immunofluorescence staining of calreticulin (CRT) after treatment with different temperatures in U14 cells. (**B)** Amount of released ATP in the medium supernatant of U14 cells frozen in refrigerators at −20 °C, −40 °C or −80 °C for 10 min or 20 min and then put at 37 °C for 10min. The black dotted line represents the concentration of ATP released by U14 cells under normal culture (37 °C). (**C)** Schematic diagram for murine U14 bilateral subcutaneous cervical cancer model and the experimental workflow. (**D)** Temperature-time curve at the center of the cryoablation probe. (**E)** Representative images of the primary tumors at day 12 and 24 after cryoablation. (**F)** Represent images of PD-L1 immunohistochemistry of the secondary tumors. (**G)** Statistical diagram of PD-L1 scores (*n* = 4/group). (**H)** The correlations between PD-L1 (CD274) and CD8A in TCGA cervical squamous cell carcinoma data (*n* = 253) (Pearson *r* = 0.4924, *P*<0.001). **(I-J)** Kaplan–Meier analysis of overall survival (OS) and progression-free survival (PFS) in patients with cervical squamous cell carcinoma in TCGA data according to the expression of PD-L1 (CD274). (**K)** Represent images of CD8 immunohistochemistry of the secondary tumors. (**L)** Quantification of the density of CD8 positive cells (*n* = 3∼4/group). (**M)** Representative flow cytometry plots and (**N)** corresponding quantitative analysis for the expression of PD-1 on the surface of CD8^+^ T cells in the secondary tumors (*n* = 3/group) (*P* = 0.02). (**O)** Representative flow cytometry plots and (**P)** corresponding quantitative analysis for the expression of PD-1 on the surface of CD4^+^ T cells in the secondary tumors (*n* = 3/group) (*P* = 0.042). The data are presented as the Mean ± SEM, and *P* values were derived from t tests.

Immunogenic cell death (ICD) may be one of the mechanisms leading to abscopal effect [16]. Therefore, we assessed two important DAMP molecules, the exposure of calreticulin (CRT) and the secretion of adenosine triphosphate (ATP). As shown in Figs. 1A and S1, cold treatments triggered more CRT exposure on U14 cells compared to 37 °C. Similarly, low temperature treatment significantly enhanced ATP secretion from treated tumor cells (Fig. 1B). These data indicate that cold treatment can promote DAMPs expression and induce potent ICD.

Using the aforementioned murine bilateral subcutaneous cervical cancer model, we undertook cryoablation on left-sided tumors (the primary tumors) (Figs. 1C and S2A). The lowest temperature at the freezing center can reach −167.7 °C and the fastest cooling rate during the rapid cooling stage can reach up to 366 °C/min, which can meet the requirements of snap-freezing (Fig. 1D). During the thawing phase, the frozen tissue was thawed slowly at room temperature. As shown in Fig. 1E, all the primary tumors achieved complete ablation, and the wound healed well after ablation. This indicates that cryoablation not only exhibits excellent local control but also facilitates postoperative tissue repair.

Anti-PD-1/PD-L1 immunotherapy might be more effective in patients who express PD-L1 and contain sufficient tumor-infiltrating PD1^+^ T cells (especially CD8^+^T cells) [17,18]. We found that cryoablation of the primary tumor can significantly increase the expression of PD-L1 in the secondary tumors (Fig. 1F and 1G). Through further analysis of cervical squamous cell carcinoma data in the TCGA database (*n* = 253), the expression of PD-L1 (CD274) was positively correlated with CD8A (Fig. 1H). Additionally, the high expression of PD-L1 was associated with better clinical prognosis in patients with cervical squamous cell carcinoma (Fig. 1I and 1J). IHC staining of the secondary tumors revealed the percentage of CD8^+^ T cell exhibited a significant increase at day 12 after cryoablation (Fig. 1K and 1L). We also found that cryoablation of the primary tumor could significantly increase the expression of PD-1 on CD8^+^T cells and CD4^+^ T cells in the secondary tumors (Fig. 1M, 1N, 1O and 1P).

Therefore, cryoablation of the primary tumor can not only increase the expression of PD-L1, but also promote the infiltration of CD8^+^ T cells and increase PD-1 expression on the surface of CD8^+^ T cells and CD4^+^ T cells within the secondary tumor. These changes provided critical condition for anti-PD-1/PD-L1 immunotherapy to exert their anti-tumor effect. Based on these, we postulated that anti-PD-1 immunotherapy in combination with cryoablation would further improve antitumor immunity.

### Combination therapy with cryoablation and anti-PD-1 antibody can produce synergistic anti-tumor effects

**Fig. 2:**
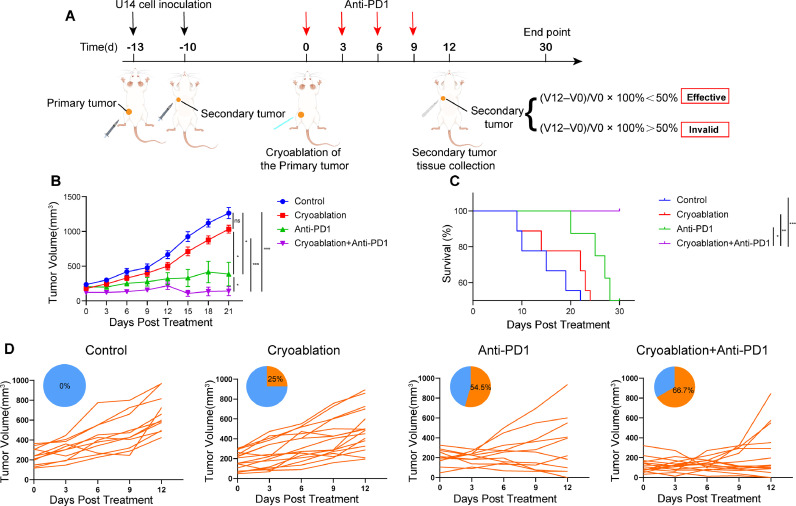
**Combination therapy with cryoablation and anti-PD-1 antibody can produce synergistic anti-tumor effects. (A)** Schematic diagram for murine U14 bilateral subcutaneous cervical cancer model and the experimental workflow. (**B)** The secondary tumor growth curve of different experimental groups (*P < 0.05, **P < 0.01, *** *P*< 0.001, ns: *P* > 0.05, not significant). (**C)** Kaplan-Meier survival curves of mice in different treatment groups. Cryoablation+Anti-PD1 VS Control, *P* <0.001; Cryoablation+Anti-PD1 VS Cryoablation, *P* = 0.003; Cryoablation+Anti-PD1 VS Anti-PD1, *P* = 0.036, P values were calculated with log-rank tests. (**D)** Combination therapy resulted in a higher therapeutic response rate. Cryoablation+Anti-PD1 VS Control, *P* = 0.001; Cryoablation VS Control, *P* = 0.123; Anti-PD1 VS Control, *P* = 0.012, *P* value according to the Fishers exact test.

By using the aforementioned mouse model (Figs. 2A and S2), we found that cryoablation of the primary tumor could partially impair the secondary tumor growth, although not statistically significant (Fig. 2B). Combination therapy with cryoablation and anti-PD-1 antibody demonstrated more pronounced inhibition of the secondary tumor growth than the other groups (Fig. 2B). In addition, combination therapy can significantly extend the survival time of tumor-bearing mice (Fig. 2C).

Further analysis of the therapeutic response rates, our data showed that compared with the control group, both cryoablation and anti-PD-1 antibody monotherapy exhibited some degree of efficacy in reducing the growth of secondary tumors, the therapeutic response rates were 25% and 54.5%, respectively. Combination therapy could further improve the therapeutic response rate (66.7%) (Fig. 2D). In summary, these results illustrate that cryoablation could synergize with anti-PD-1 antibody, thus cause more powerful anti-tumor efficacy.

### Combination therapy enhances infiltrations and functions of T cells in distant tumors

**Fig. 3:**
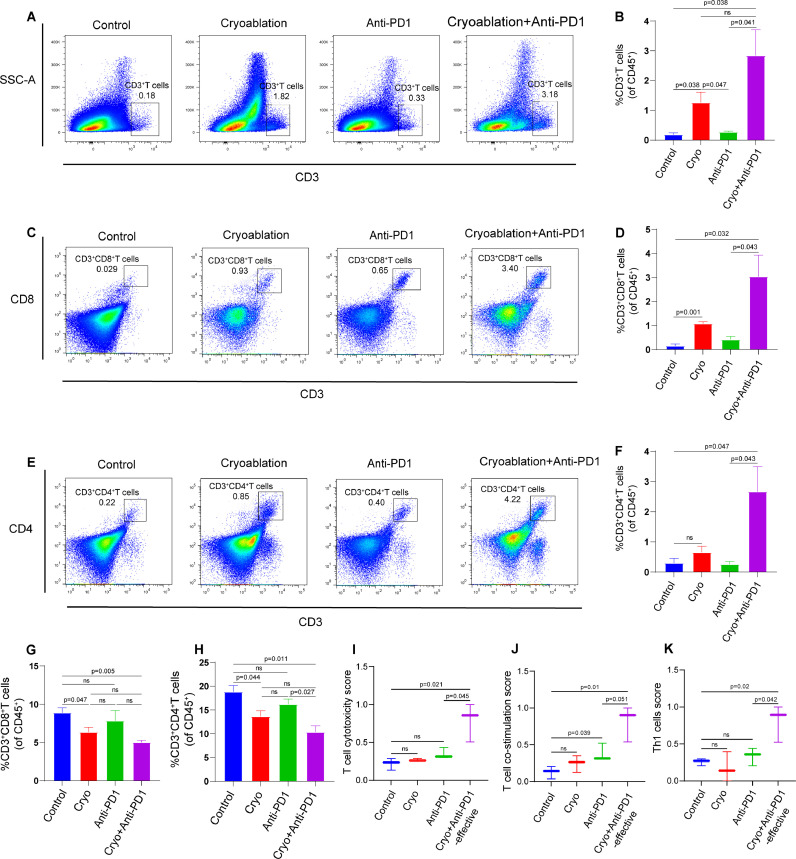
**Combination therapy increases the infiltration and anti-tumor function of T cells in distant secondary tumors. (A)** Representative flow cytometry plots and (**B)** corresponding quantitative analysis for the infiltration of T cells in the secondary tumors (*n* = 3/group). (**C)** Representative flow cytometry plots and (**D)** corresponding quantitative analysis for CD8^+^ T cells proportion in the secondary tumors (*n* = 3/group). (**E)** Representative flow cytometry plots and (**F)** corresponding quantitative analysis for CD4^+^ T cells proportion in the secondary tumors (*n* = 3/group). (**G-H)** Percentage of CD8^+^ T cells and CD4^+^ T cells in the spleen of different experimental groups analyzed by flow cytometry (*n* = 3/group). (**I-K)** T cell cytotoxicity score, T cell co-stimulation score and Th1 cells score in secondary tumors of different experimental groups were analyzed by using ssGSEA. The data are presented as the Mean ± SEM, and *P* values were derived from t tests.

To explore the impact of combination therapy on the tumor immune microenvironment (TIME), we first analyzed the infiltrating T cells in the secondary tumors by flow cytometry. Our experimental results suggested that cryoablation could promote T cells infiltration in the secondary tumors (Fig. 3A and 3B). In particular, the proportion of infiltrating CD8^+^ T cells increased significantly (Fig. 3C and 3D). Combination therapy with cryoablation and anti-PD-1 antibody exhibited a notably higher frequency of infiltrating CD8^+^ T cells than the other groups (Fig. 3C and 3D). Further examination of CD4^+^ T cell populations also indicated that combination therapy effectively enhanced the infiltration of CD4^+^ T cells into the secondary tumors (Fig. 3E and 3F).

The spleen serves as the anatomical location for the settlement of mature T cells, and T cells settled here differentiate into effector T cells after receiving antigens presented by antigen presenting cells (APCs) [19]. T cells in the spleen can infiltrate into non-lymphoid tissues in an antigen-specific manner [19]. Our study revealed a decrease in the count of splenic CD8^+^ and CD4^+^ T cells in both cryoablation group and combination therapy group, as compared to the isotype control group (Fig. 3G and 3H). However, anti-PD-1 antibody monotherapy did not have a significant impact on the levels of CD8^+^ T cells and CD4^+^ T cells in the spleen (Fig. 3G and 3H). Consequently, we can infer that cryoablation, either alone or in combination with anti-PD-1 antibody, does not stimulate the proliferation and activation of T cells systemically. Cryoablation appears to play a crucial role in facilitating the infiltration of T cells into the tumor microenvironment.

Effective anti-tumor immune response requires not only the infiltration of immune effector cells into the tumor, but also these cells can exert their function. By using ssGSEA, we found that combination therapy could significantly promote the cytotoxic effect of T cells, contribute to T cell activation, and increase the content of Th1 cells within the distant secondary tumors (Fig. 3I, 3J and 3K). In contrast, cryoablation failed to induce a substantial cytotoxic T cell response in the secondary tumors, only marginally augmenting T cell activation (Fig. 3I, 3J and 3K). Collectively, our results demonstrate that local cryoablation can help improve the infiltration of T cells, especially CD8^+^ T cells in distant tumors. Cryoablation combined with anti-PD-1 antibody can not only promote the infiltration of CD8^+^ T cells, but also significantly activate the infiltrated CD8^+^ T cells in distant tumors.

### Combination therapy alters the percentages of myeloid immune subsets (TAMs, MDSCs and DCs) in distant tumors

**Fig. 4:**
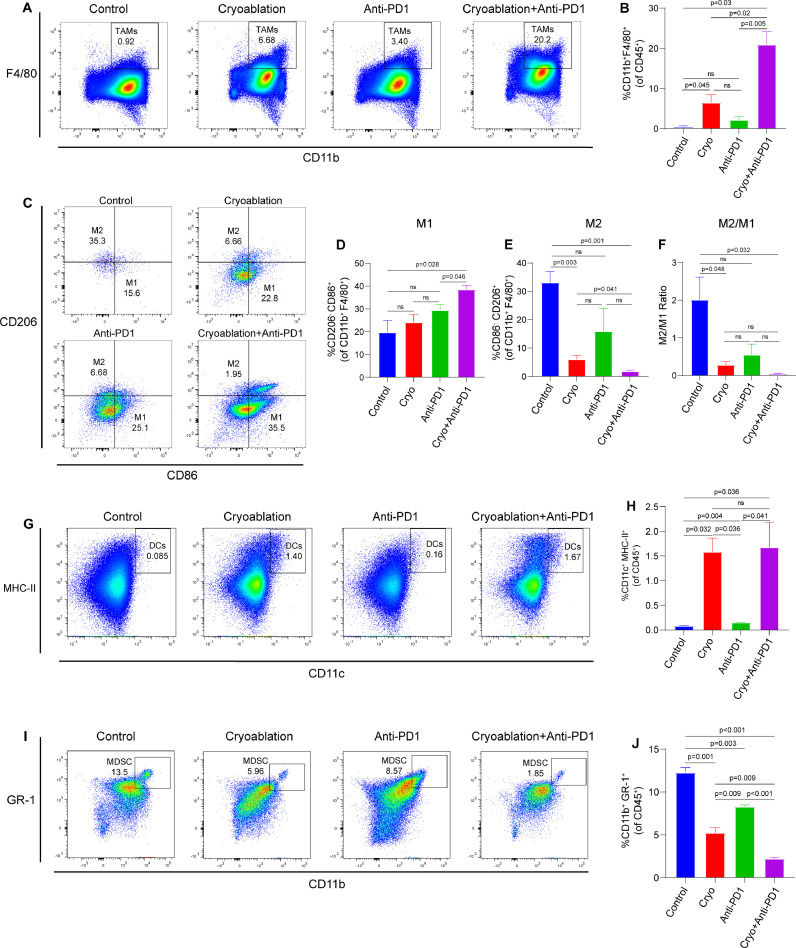
**Combination therapy alters the percentages of myeloid immune subsets in distant tumors. (A)** Representative flow cytometry plots and (**B)** corresponding quantitative analysis for the infiltration of TAMs in the secondary tumors (*n* = 3/group). (**C)** Representative flow cytometry plots for the infiltration of M1 and M2 like TAMs in the secondary tumors. (**D-F)** Quantitative analysis of the flow cytometry results about the infiltration of M1-like TAMs, M2-like TAMs and the ratio of M2/M1 (*n* = 3/group). (**G)** Representative flow cytometry plots and (**H)** corresponding quantitative analysis for the infiltration of DCs in the secondary tumors (*n* = 3/group). (**I)** Representative flow cytometry plots and (**J)** corresponding quantitative analysis for the infiltration of MDSCs in the secondary tumors (*n* = 3/group). The data are presented as the Mean ± SEM, and *P* values were derived from t tests.

Tumor-associated macrophages (TAMs) are highly plastic inflammatory cells. There are two main phenotypes: M1-like TAMs with tumor-killing functions and M2-like TAMs with immune-suppressive functions [20,21]. Studies have suggested that the reduction of M2/M1 ratio in tumor microenvironment can enhance the anti-tumor immune response by promoting the infiltration and activation of cytotoxic T cells [21,22]. We found that cryoablation could significantly increase the infiltration of TAMs, decrease the proportion of M2-like TAMs and reduce the ratio of M2/M1 in the secondary tumors in comparison to isotype control. Combination therapy further enhanced the infiltration of TAMs, reduced the percentage of M2-like TAMs, upregulated the population of M1-like TAMs and significantly decreased the M2/M1 ratio in the secondary tumors (Fig. 4A–F). The observed effect appeared to be mainly mediated by cryoablation, as anti-PD-1 antibody monotherapy only slightly affected TAMs and their polarization, and all differences were not statistically significant.

Dendritic cells (DCs) are highly efficient APCs that serve as crucial intermediaries between innate and adaptive immunity, tumor infiltration of mature DCs enhances immune activation and the recruitment of antitumor effector cells [23,24]. Our study revealed that cryoablation, whether used as monotherapy or in combination with anti-PD-1 antibody, significantly augmented DCs infiltration into the secondary tumors (Fig. 4G and 4H). This suggests that the observed alterations in DCs within the combination group primarily arise from the effects of cryoablation.

Myeloid‐derived suppressor cells (MDSCs) are important immunosuppressive cells that assume a pivotal function in tumor progression by suppressing innate and adaptive immunity [25,26]. We found that both cryoablation and the combination therapy with cryoablation and anti-PD-1 antibody could induce a remarkable reduction of MDSCs in the secondary tumors compared with isotype control (Fig. 4I and 4J).

Collectively, combination therapy with cryoablation and anti-PD-1 antibody alters the percentages of myeloid immune subsets (TAMs, MDSCs and DCs) in distant secondary tumors, including increase the infiltration of TAMs and DCs, reduce M2/M1 ratio and the percentage of MDSCs.

### Combination therapy modulates intratumor chemokine milieu of the distant tumors

One of the main factors that promotes directional migration of immune cells is the production of chemokines, which can guide immune cells from circulation into the tumor microenvironment. We found that cryoablation combined with anti-PD-1 antibody could enhance the production of multiple proinflammatory chemokines in distant tumors, especially CCL2, CCL5, CXCL9 and CXCL10 (Fig. S4A and S4B). The expression of these four chemokines showed a positive correlation not only with CD8a, but also with the infiltration of innate immune cells into the tumors (DCs and TAMs) (Fig. S4C, S4D and S4E).

Consistent with the experimental results, by analyzing the TCGA database, we also found that the expression of CCL2, CCL5, CXCL9, and CXCL10 was significantly positively correlated with the expression of CD8A and the infiltration of DCs and TAMs in patients with cervical squamous cell carcinoma (*n* = 253) (Fig. S4F, S4 G and S4H).

Taken together, combination therapy can significantly promote the expression of chemokines related to the infiltration of CD8^+^ T cells, DCs, and TAMs in distant tumors, which explains why the distant secondary tumors of mice treated with combination therapy had higher infiltration of CD8^+^ T cells, DCs, and TAMs.

### Cryoablation can exert an immunomodulatory effect in distant tumors

**Fig. 5:**
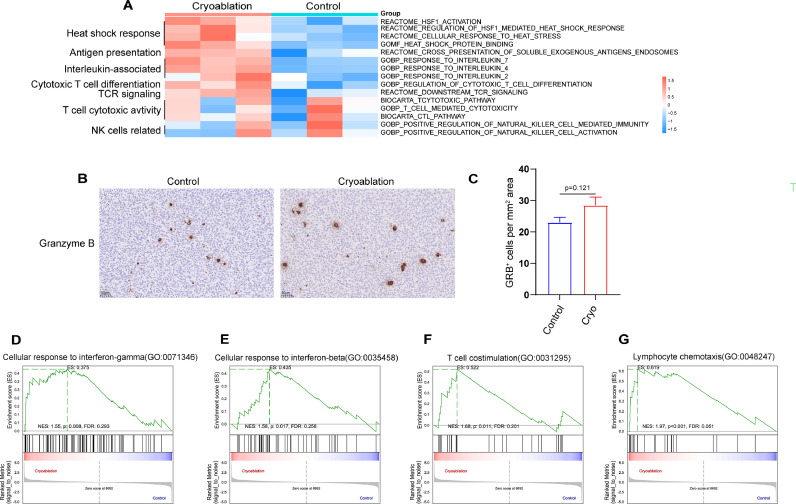
**Cryoablation can exert an immunomodulatory effect in distant tumors.** (**A)** GSVA was exploited to analyze the difference of molecular pathways between the cryoablation group and control group. (**B)** Representative Granzyme B immunohistochemical results in the secondary tumors of different treatment groups (Control VS Cryoablation) and (**C)** corresponding quantitative analysis for the density of Granzyme B positive cells. The data are presented as the Mean ± SEM, and *P* values were derived from t tests. (**D-G)** GSEA plots showing significant enrichment of IFN-γ and IFN-β signaling pathway, T cell co-stimulation signaling pathway and lymphocyte chemotaxis signaling pathway in the cryoablation group.

To dissect the impact of focal cryoablation treatment on distant tumor immune landscape, we quantified the enrichment results of different molecular pathways using GSVA. We found that cryoablation induced a significant upregulation of signaling pathways related to heat shock response, antigen presentation, cytotoxic T lymphocyte differentiation and T cell receptor (TCR) in secondary tumors (Fig. 5A). We also found several proinflammatory interleukin-related signaling pathways were highly enriched in the cryoablation group, including IL-2, IL-4 and IL-7. However, cryoablation did not significantly activate T cell toxicity related pathway and NK cell activity related pathway (*P* > 0.05). Through immunohistochemical analysis of Granzyme B (Gra B) expression, which serves as an indicator of immune activation[27], we found no statistically significant difference in the expression of Gra B in the secondary tumors between cryoablation group and control group (Fig. 5B and 5C). That is to say, although cryoablation could promote CD8^+^ T cells infiltration in the secondary tumors, the tumor-killing action triggered by cryoablation monotherapy is comparatively weak.

By analyzing the differentially expressed genes between the cryoablation group and the control group, we found that cryoablation of the primary tumor significantly increases the expression levels of several chemokines in the secondary tumors, including Ccl2, Ccl6, and Ccl7. Notably, these chemokines show a significant positive correlation with the infiltration of CD8^+^ T cells, DCs, and TAMs (Fig. S5). This partially explains why cryoablation promotes the infiltration of various immune cells in distant tumors.

Additionally, by using GSEA, we found that pathways related to T cell co-stimulation, lymphocyte chemotaxis, cellular response to interferon (IFN)-γ and IFN-β were positively enriched in cryoablation group compared with isotype control (Fig. 5D–G). Collectively, our results indicate that cryoablation triggered an inflammatory process in the distant tumors, turned the TME from immunologically “cold” into “hot”, which can significantly enhance the sensitivity of anti-PD-1 immunotherapy.

### Combination therapy facilitates multiple aspects of anti-tumor immune pathways compared with anti-PD-1 antibody monotherapy

**Fig. 6:**
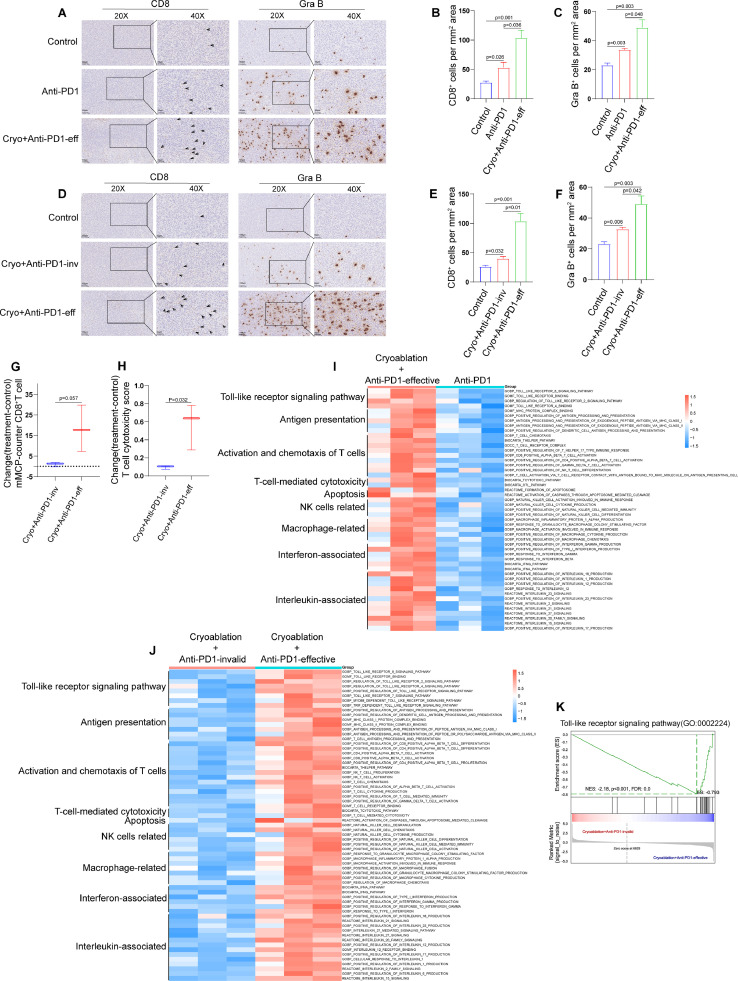
**Cryoablation combined with anti-PD-1 antibody facilitates multiple aspects of anti-tumor immune pathways.** (**A)** Representative CD8 and Granzyme B immunohistochemical results in the secondary tumors of different treatment groups and (**B-C)** corresponding quantitative analysis. (**D)** Representative CD8 and Granzyme B immunohistochemical results in the secondary tumors of different treatment groups and (**E, F)** corresponding quantitative analysis. (**G)** The abundance of CD8^+^ T cells in the secondary tumors was evaluated by using mMCP-counter. (**H)** T cell cytotoxicity score in secondary tumors was calculated by ssGSEA. (**I)** GSVA was exploited to analyze the difference of molecular pathways between the effective combination therapy and anti-PD-1 antibody monotherapy group. (**J)** GSVA was exploited to analyze the difference of molecular pathway between the invalid group and effective group of combination therapy. (**K)** GSEA analysis showed that Toll-like receptor signaling pathway was significantly inhibited in the invalid group of combination therapy. The data are presented as the Mean ± SEM, and *P* values were derived from t tests.

To explain why combination therapy with cryoablation and anti-PD-1 antibody can exert better anti-tumor effect than anti-PD-1 antibody alone, we first analyzed the expression of CD8 and Gra B in the secondary tumors by immunohistochemistry. Our experimental results suggested that combination therapy could significantly promote CD8^+^ T cells infiltration and Gra B expression in the secondary tumors compared with anti-PD-1 antibody monotherapy (Fig. 6A–C).

Further analyses revealed that combination therapy yields a notable augmentation in various anti-tumor immune pathways compared with anti-PD-1 antibody monotherapy by using GSVA. These pathways encompass tumor antigens presentation, T cell activation and chemotactic capacity, T cell mediated cytotoxicity, Toll-like receptor related signal pathways, NK cell and macrophage related immune response as well as apoptosis related signal pathways (Fig. 6I). This result further confirms that cryoablation combined with anti-PD-1 antibody resulted in overall improvement of anti-tumor immune pathways and has more therapeutic advantages compared with anti-PD-1 antibody monotherapy.

### The inadequate activation of the toll-like receptor signaling pathway may be one of the reasons for the poor response of combination therapy

Although cryoablation combined with anti-PD-1 antibody can significantly control tumor growth and stimulate anti-tumor immune responses, there is still a subset of mice that do not respond to the treatment. Therefore, it is crucial to comprehend the underlying factors responsible for this resistance in order to identify more appropriate combination therapies.

We found that the infiltration of CD8^+^ T cells and the expression of Gra B in the secondary tumors in invalid combination therapy group were significantly lower than those in effective combination therapy group (Fig. 6D, 6E and 6F). The bioinformatics analysis of RNA-seq data also found that there were significant differences in CD8^+^ T cell infiltration and T cell-mediated cytotoxicity in the secondary tumors between the effective group and the invalid group of combination therapy (Fig. 6G and 6H). We also found that the significant differences in pathways between the effective and invalid groups of combination therapy focused on multiple anti-tumor immune response steps such as tumor antigens presentation, T cell activation and chemotactic capacity, NK cell and macrophage-related immune response as well as apoptosis-related signal pathway (Fig. 6J).

In particular, we found that Toll-like receptor-related signal pathway was significantly down-regulated in the invalid group of combination therapy. The GSEA analysis came to the same conclusion (Fig. 6K). The inadequate activation of the Toll-like receptor signaling pathway may be one of the reasons for the poor response of cryoablation combined with anti-PD-1 antibody. Therefore, suitable TLR agonist may be used to further improve the efficacy of the combination therapy (cryoablation + anti-PD-1 antibody).

## Discussion

In this study, we demonstrated that cryoablation combined with anti-PD-1 immunotherapy synergistically enhances antitumor immunity, resulting in superior tumor control and survival rates in cervical cancer mouse models. First, cryoablation of the primary tumor not only upregulates the expression of PD-L1, but also facilitates the infiltration of CD8^+^ T cells and increases the expression of PD-1 on the surfaces of CD8^+^ T cells and CD4^+^ T cells within the secondary tumor. These alterations create a crucial environment for anti-PD-1 immunotherapy to exert its anti-tumor efficacy. Second, combination therapy (cryoablation + anti-PD-1 antibody) leads to a conversion of the distant tumor TME from immunosuppressive to immunostimulatory, favoring the infiltration of CD8^+^T cells, CD4^+^T cells, DCs and M1-like TAMs, enhancing multiple aspects of the antitumor immune response while simultaneously reducing immunosuppressive populations such as M2-like TAMs and MDSCs. The decrease in the content of these two types of immunosuppressive cells in the tumor microenvironment removes the obstacles to the anti-tumor immune response. The findings of our study offer preclinical evidence supporting the potential therapeutic application of cryoablation in combination with anti-PD-1 antibody for the treatment of patients diagnosed with advanced/recurrent cervical cancer.

Cryoablation can effectively destroy primary or more manageable tumors, thereby facilitating the release of a cascade of tumor antigens into circulation. Specifically, compared to heat-based ablations, cryoablation can better preserve the immunogenicity of tumor antigens [28]. These tumor antigens possess the capacity to serve as“*in situ*”cancer vaccines to activate the anti-tumor immune response [29]. Cryoablation of the primary tumor may even induce “abscopal effect’’, that is, cryoablation can induce the reduction or disappearance of cancerous foci in non-frozen sites [10]. This phenomenon holds significant importance in tumor therapy, as it effectively diminishes the likelihood of tumor recurrence and metastasis. However, in most cases, the anti-tumor immune response triggered by cryoablation is relatively weak, especially for advanced tumors with high tumor burden. Some studies argue that it may be due to the pre-existing immunosuppressive tumor microenvironment, including inhibitory immune checkpoints [30,31]. Consequently, it becomes imperative to complement cryoablation with suitable immunotherapy to augment the anti-tumor immune response.

The emergence of immunotherapy represented by ICIs has revolutionized the treatment of cancer, but not all patients respond to the treatment. The effective rate of ICIs alone is only about 20% [32]. The possible mechanistic basis of tumor immunotherapy resistance includes loss of tumor-specific antigens, low infiltration of antitumor immune cells in tumor microenvironment, rapid expression of multiple immune checkpoint ligands and the infiltration of immunosuppressive immune cells [33]. In particular, the role of ICIs requires the existence of immune recognition and immune response to tumors, so“cold tumors”with low immunogenicity and low immune cells infiltration are particularly resistant to immunotherapy. Therefore, combined with therapeutic methods that can induce tumor immunogenicity and immune cell infiltration in tumor has become an important method to increase the efficacy of immunotherapy. Cryoablation can be a potential option to improve the therapeutic effect of immunotherapy.

An effective and durable anti-tumor immune response requires the procedural realization of three important steps: (1) acquisition or exposure of tumor antigens; (2) tumor antigen presentation and activation of immune cells; (3) intra-tumoral infiltration of immune cells and their attack on tumor cells. Correspondingly, tumor immunotherapy also consists of three key components: (1) promoting the acquisition/exposure of tumor antigens; (2) promoting antigens presentation and immune activation; (3) relieving the inhibition of T cells. The acquisition or exposure of tumor antigens plays an“ignition”role in activating the anti-tumor immune response, cryoablation can fulfil such a role. Our study also found that cryoablation could exert immunomodulatory effects, thereby reshaping the immune microenvironment of distant tumors. Cryoablation could cause the release of DAMP signals such as surface exposure of CRT, release of ATP and HSPs. These DAMPs can not only enhance the phagocytosis of antigen-presenting cells (APCs) [34], but also induce the expression of co-stimulatory molecules on APCs [35,36], which can more effectively promote antigen presentation and immune cell activation.

The mechanisms by which cryoablation of primary tumors enhances immune infiltration in secondary tumors remain incompletely understood. Previous studies have suggested that alterations in the expression of immune cell recruitment-associated chemokines within the tumor microenvironment might play a pivotal role in this process [37]. Furthermore, studies have suggested that type I IFN-dependent antitumor immune responses are closely associated with the recruitment of CD8^+^ T cells to tumor sites, as well as the expression of proinflammatory cytokines and chemokines within the tumor microenvironment [38]. Our research has similarly demonstrated that cryoablation of the primary tumor significantly enhances the expression of various chemokines associated with immune cell recruitment in the secondary tumor. Additionally, we observed a substantial upregulation of IFN-related signaling pathways in the secondary tumor. While our findings shed light on potential mechanisms driving this immune landscape alteration, further investigation is warranted to unravel the intricate mechanisms at play.

Our research revealed that although cryoablation can promote the infiltration of CD8^+^ T cells in distant tumors, it does not trigger significant antitumor activity. One possible reason may be that the activation of IFN signaling pathway caused by sustained T cell activation can promote the expression of PD-1 and PD-L1 in TME, which leads to T cell exhaustion [[39], [40], [41], [42]]. But this process created suitable tumor immune microenvironment (TIME) for anti-PD-1/PD-L1 immunotherapy. The anti-tumor effect of anti-PD-1/PD-L1 immunotherapy is not mediated by the antibody itself, but by tumor antigen-specific T cells suppressed by PD-1/PD-L1 immune checkpoint mechanism [43,44]. Solid tumors can be classified into four types of TIME based on the expression of PD-L1 and tumor-infiltrating lymphocytes (TIL) (mainly based on CD8^+^ T cells): PD-L1-/TIL- (type I); PD-L1+/TIL+ (type II); PD-L1-/TIL+ (type III); PD-L1+/TIL- (type IV). Type II tumors are associated with a better anti-PD-1/PD-L1 immunotherapy response [45]. Our results suggest that cryoablation of the primary tumor can not only increase the expression of PD-L1, but also promote the infiltration of CD8^+^ T cells and increase PD-1 expression on the surface of CD8^+^ T cells and CD4^+^ T cells within the secondary tumor. The formation of this type II TIME provides critical conditions for anti-PD-1 immunotherapy to exert its anti-tumor effect.

## Conclusions

In conclusion, our study found that the combinational strategy of cryoablation and anti-PD-1 immunotherapy is able not only to eradicate the primary tumors directly but also to induce a series of host immune responses to destroy residual tumor cells and inhibit distant tumors. Combining cryoablation with anti-PD-1 immunotherapy could elicit a stronger immune response than either cryoablation or anti-PD-1 immunotherapy alone. The combination of cryoablation and anti-PD-1 antibody can jointly improve antitumor immune responses by potentiating the antitumor effects and reversing the immunosuppressive nature of the cervical cancer tumor microenvironment.

## Availability of data and materials

All data supporting the findings of this study are available from the corresponding authors upon reasonable request.

## Ethics approval and consent to participate

The experimental procedures with animals were approved by the Ethics Committee of International Peace Maternity & Child Health Hospital, School of Medicine, Shanghai Jiaotong University, and were performed in accordance with the institutional guidelines.

## Consent for publication

The authors give the consent for the submission of this manuscript to the *Translational Oncology* and, if accepted, to its publication in this journal.

## CRediT authorship contribution statement

**Xiaoming Yang:** Writing – original draft, Visualization, Methodology, Formal analysis. **Xiaoyan Gao:** Writing – review & editing, Software, Methodology, Formal analysis. **Chen Xu:** Visualization, Data curation. **Ting Ni:** Data curation. **Yaru Sheng:** Methodology. **Jing Wang:** Formal analysis. **Xiao Sun:** Methodology. **Jiangjing Yuan:** Visualization, Methodology. **Lin Zhang:** Writing – review & editing, Visualization, Data curation. **Yudong Wang:** Writing – review & editing, Supervision, Project administration, Funding acquisition, Conceptualization.

## Declaration of competing interest

The authors declare that they have no known competing financial interests or personal relationships that could have appeared to influence the work reported in this paper.

## Appendix Supplementary materials

Image, application 1

Image, application 2

Image, application 3

Image, application 4
