# Supplementary material for: Cryoablation synergizes with anti-PD-1 immunotherapy induces an effective abscopal effect in murine model of cervical cancer
Source: Transl Oncol. 2024 Nov 2;51:102175. doi: 10.1016/j.tranon.2024.102175 (PMC11565560; doi:10.1016/j.tranon.2024.102175)
Supplement: Supplementary file 1 [file mmc1.docx]

**Detailed information on the construction of murine model**

To explore the underlying immune response induced by cryoablation, we constructed a murine bilateral subcutaneous cervical cancer model in which U14 squamous cell carcinoma cell line was inoculated subcutaneously into the bilateral flanks of BALB/c mice. The primary tumor was inoculated subcutaneously with 5×10^6^ U14 cells in 200μl PBS on the left flank back. The secondary tumor, as distant tumor, was inoculated subcutaneously with 1×10^6^ U14 cells in 200μl PBS in the right axilla 3 days after the inoculate of the primary tumor. HE staining was performed to confirm tumor formation. Only mice with palpable tumor growth on both sides and the primary tumor volume reached approximately 200 mm^3^ can be included in this study. The mice were excluded if they died during anesthetic treatment or cryoablation.

**Cryoablation and detailed information on experimental design**

Mice were anaesthetized with 2% isoflurane. Once anesthetized, the hair overlying the primary tumor site was removed and the puncture site was disinfected with 75% alcohol. A 1.7mm-diameter cryoprobe was inserted into the center of the primary tumor, freezing for 30 s and then natural rewarming to 10°C. Mice were put on electric blanket during the entire duration of cryoablation and provided all necessary post procedural care.

When the primary tumor volume reached approximately 200 mm^3^, tumor-bearing bilateral mice were randomly divided into four experimental groups: (1) control group (untreated, n=11), (2) cryoablation group (cryoablation plus an isotype control antibody, n=16), (3) anti-PD-1 group (anti-PD-1 antibody monotherapy, n=11), and (4) combination therapy group (cryoablation plus anti-PD-1 antibody, n=15). Cryoablation was given to the primary tumor on day 0, when the primary tumor volume reached approximately 200 mm^3^. Anti-PD-1 (10 mg/kg, BioXcell) was injected intraperitoneally every 3 days, starting on day 0 after cryoablation for 30 minutes (day 0, day 3, day 6, day 9). The mice in cryoablation group received intraperitoneal injections of mouse IgG (10 mg/kg, BioXcell, day 0, day 3, day 6, day 9). Mice were observed daily for tumor formation and survival. The tumor size was measured every 3 days by using vernier caliper and tumor volumes (mm^3^) were calculated according to the following formula: tumor volume (mm^3^) = 0.5×L×W^2^(L, the longest diameter; W, the diameter perpendicular to the longest diameter). At day 12, some mice from each group were sacrificed for analysis. Survival analysis was continued as independent experiments for indicated days. The total study period was 30 days and mice were euthanized when tumor volume exceeded 1500 mm^3^, or at day 30. The therapeutic efficacy was evaluated by quantifying the percentage alteration in secondary tumor volume on day 12 post-treatment compared to the volume at the start of therapy. Effective treatment was defined as (V12–V0)/V0 × 100% <50%, and invalid treatment was defined as (V12–V0)/V0 × 100% ＞50%. The number of mice euthanized for harvesting tumors for experimental analysis on Day 12 was as follows: 3 mice from the control group, 3 mice from the cryoablation group, 3 mice from the anti-PD-1 group, and 6 mice from the combination therapy group (with 3 in the effective response subgroup and 3 in the invalid response subgroup). The mice chosen for experimental analyses were selected randomly from the control, cryoablation, and anti-PD-1 antibody groups, with 3 mice from each group. In the combination therapy group, mice were divided into effective and invalid response groups based on their treatment responses, with 3 mice selected from each subgroup.

**Detailed immunohistochemical staining scoring method**

PD-L1 expression was analyzed according to immunoreactive score. Five visual fields were randomly selected under high magnification, and score them based on the percentage of positive cells and the staining intensity. The final immunoreactive score of PD-L1 under this field was obtained by multiplying the two scores (staining intensity × percentage of positive cells). The average score of the five visual fields was the final PD-L1 immunoreactive score of the slice. The staining intensity was scored as 0 (no tumor cell staining), 1 (weak staining, light yellow), 2 (moderate staining, yellow brown), and 3 (strong staining, brown). The percentage of positive cells was graded on the following scale: 1 (staining range, ≤25%), 2 (staining range, 26-50%), 3 (staining range, 51-75%), 4 (staining range, ＞75%). The immunohistochemical results of CD8 and Granzyme B were expressed as density (number of positive cells/mm^2^). Five visual fields were randomly selected from each section to count the number of marker-positive cells and the obtained cell count was divided by the area to generate the cell density (cells/mm^2^).
