# Supplementary material for: Cryoablation synergizes with anti-PD-1 immunotherapy induces an effective abscopal effect in murine model of cervical cancer
Source: Transl Oncol. 2024 Nov 2;51:102175. doi: 10.1016/j.tranon.2024.102175 (PMC11565560; doi:10.1016/j.tranon.2024.102175)
Supplement: Supplementary file 2 [file mmc2.docx]

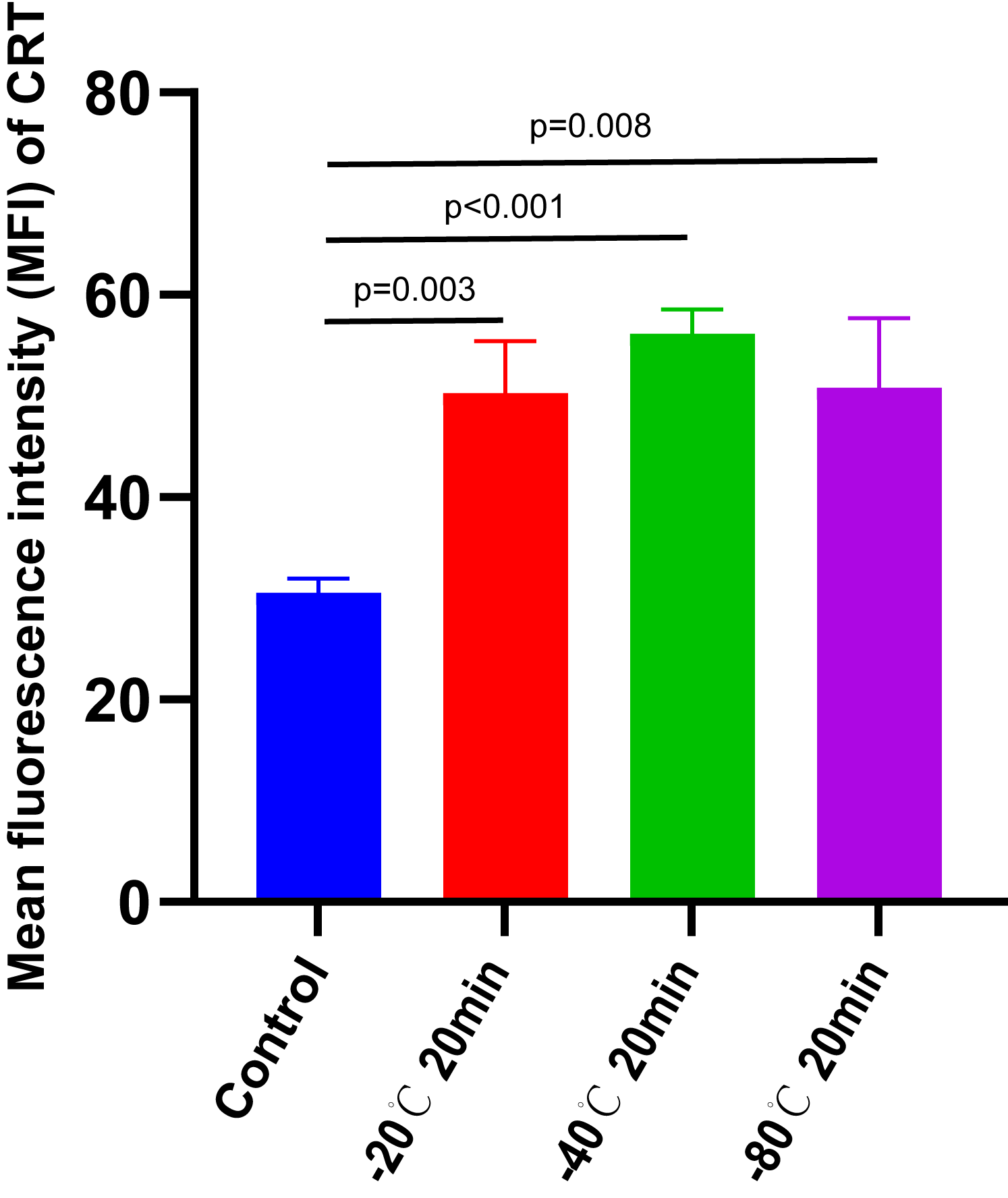


**Supplementary Figure 1. Quantitative calculation of mean fluorescence intensity(MFI) of CRT.**

**.**


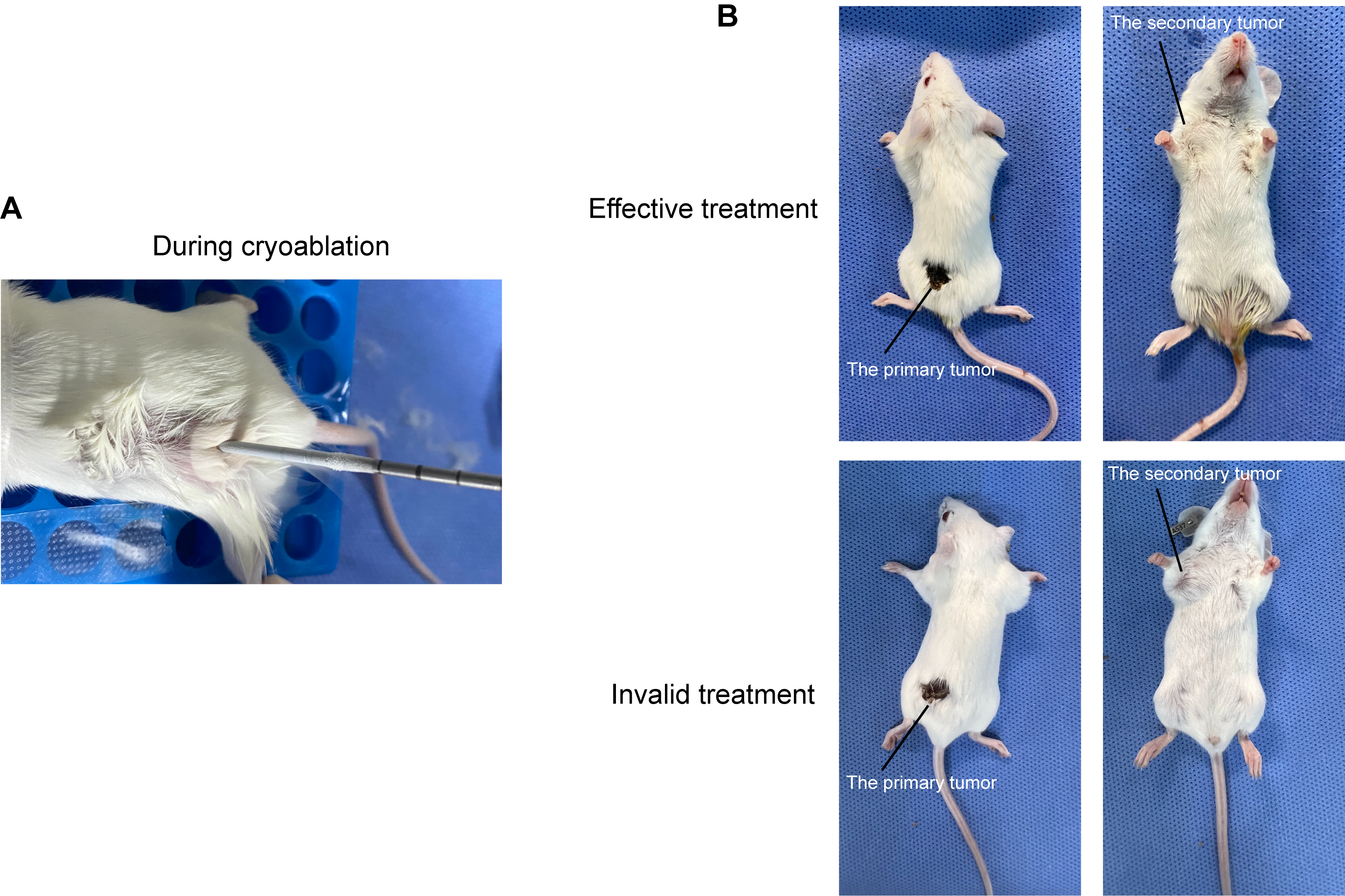


**Supplementary Figure 2. Observation during and after cryoablation. (A)** Representative image of the cryoablation process for the primary tumor. **(B)** Representative images of the primary and secondary tumors in mice at day 12 post combination therapy. The images depict examples of effective response versus invalid response to treatment, showcasing notable differences in the secondary tumor appearances.


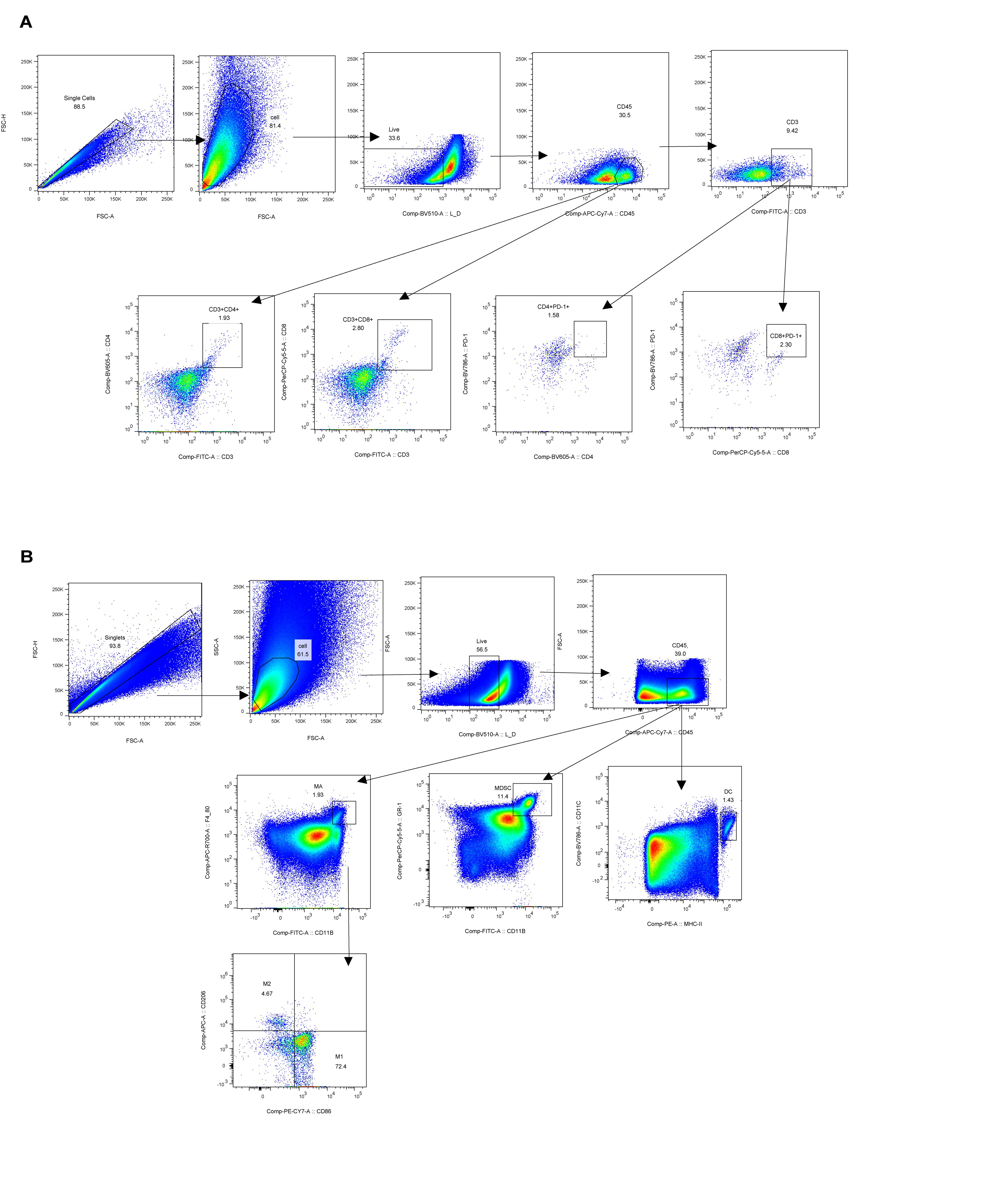


**Supplementary Figure 3. Flow cytometry gating strategy.**


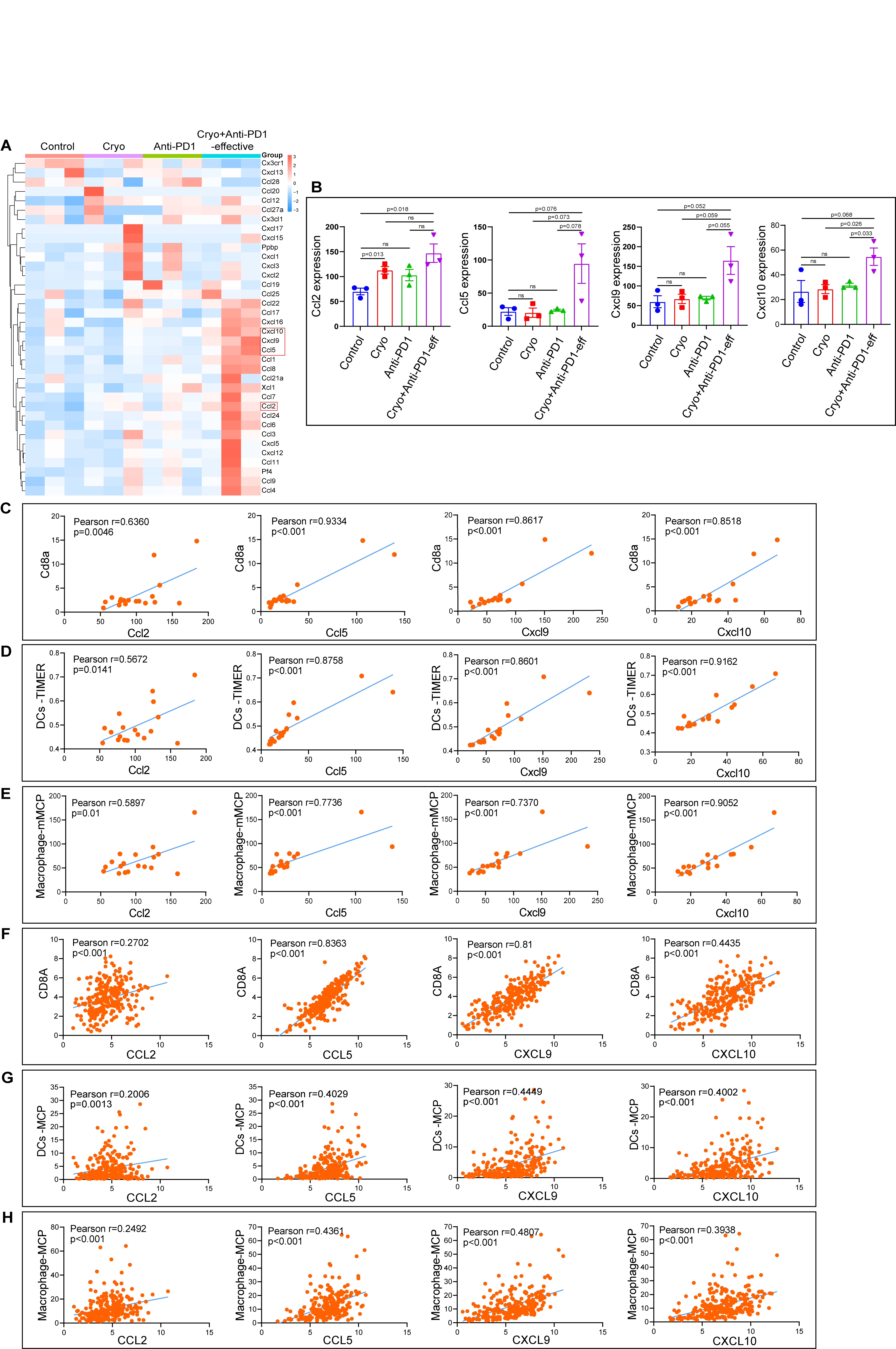


**Supplementary Figure 4. The combination of cryoablation and anti-PD-1 antibody modulates intratumor chemokine milieu of the distant tumors**. **(A)** Heatmap showing the expression of chemokines in the secondary tumors in different experimental groups (red represents high expression and blue represents low expression). **(B)** The expression of CCL2, CCL5, CXCL9, and CXCL10 in the secondary tumors in different experimental groups. The data are presented as the Mean ± SEM, and P values were derived from t tests. **(C)** Correlation analyses of CD8a expression with that of Ccl2, Ccl5, Cxcl9, and Cxcl10 in the secondary tumors. **(D)** Correlation analyses of the infiltration of DCs (estimated using the TIMER algorithm) with that of Ccl2, Ccl5, Cxcl9, and Cxcl10 expression in the secondary tumors. **(E)** Correlation analyses of the infiltration of macrophage (estimated using the mMCP counter algorithm) with that of Ccl2, Ccl5, Cxcl9, and Cxcl10 expression in the secondary tumors. **(F-H)** Scatter plots showing the correlation between CCL2, CCL5, CXCL9, CXCL10 expression levels and CD8A expression, DCs (estimated using the MCP counter algorithm), macrophage (estimated using the MCP counter algorithm) in patients with cervical squamous cell carcinoma in TCGA database.


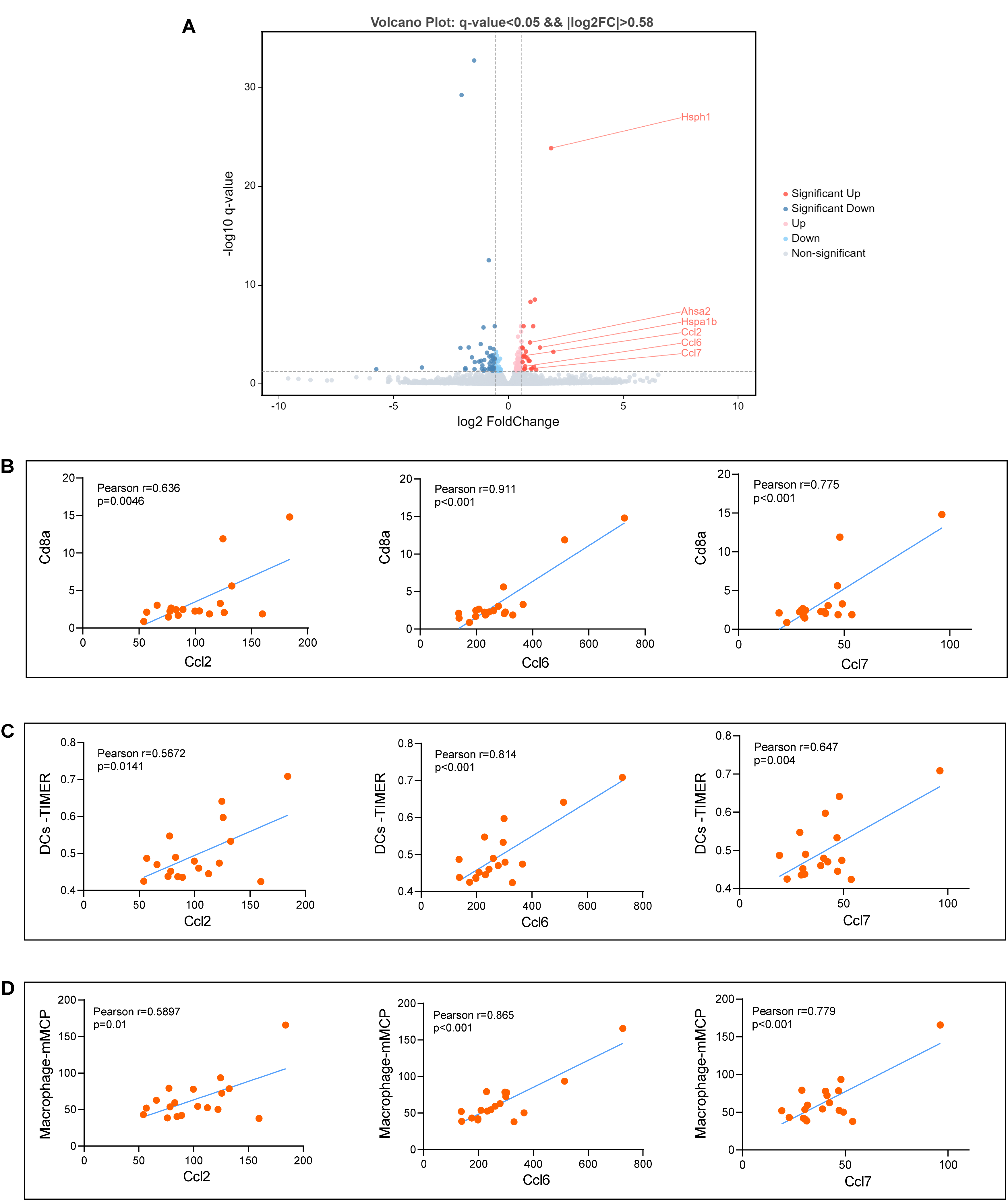


**Supplementary Figure 5. Cryoablation promotes the expression of a variety of chemokines in distant tumors. (A)** Volcano plots of differentially expressed mRNAs (cryoablation group vs control group). The red dots represent upregulated mRNAs, and the blue dots represent downregulated mRNAs. **(B)** Correlation analyses were conducted to examine the relationship between the expression of CD8a and the expression levels of Ccl2, Ccl6, and Ccl7 in the secondary tumors. **(C)** Correlation analyses were conducted to examine the relationship between the infiltration of DCs (estimated using the TIMER algorithm) and the expression levels of Ccl2, Ccl6, and Ccl7 in the secondary tumors. **(D)** Correlation analyses were conducted to examine the relationship between macrophage infiltration (estimated using the mMCP counter algorithm) and the expression levels of Ccl2, Ccl6, and Ccl7 in the secondary tumors.
