# Supplementary material for: Cryoablation synergizes with anti-PD-1 immunotherapy induces an effective abscopal effect in murine model of cervical cancer
Source: Transl Oncol. 2024 Nov 2;51:102175. doi: 10.1016/j.tranon.2024.102175 (PMC11565560; doi:10.1016/j.tranon.2024.102175)
Supplement: Supplementary file 3 [file mmc3.docx]

Table S1. Antibodies for FACS

| **Antibodies** | **Fluorochrome** | **Source** |
| --- | --- | --- |
| anti-mouse CD279 (PD-1) Antibody（748264） | BV786 | BD Pharmingen |
| anti-mouse CD8a Antibody（566409） | BB700 | BD Pharmingen |
| anti-mouse CD3e Antibody（553061） | FITC | BD Pharmingen |
| anti-mouse CD45 Antibody（557659） | APC/Cyanine7 | BD Pharmingen |
| anti-mouse CD4 Antibody（563151） | Brilliant Violet 605 | BD Pharmingen |
| anti-mouse F4/80 Antibody（565787） | APC-R700 | BD Pharmingen |
| anti-mouse CD11b Antibody（557396） | FITC | BD Pharmingen |
| anti-mouse CD206 Recombinant Antibody（141708） | APC | Biolegend |
| anti-mouse CD86 Antibody（560582） | PE/Cyanine7 | BD Pharmingen |
| anti-mouse Ly-6G and Ly-6C Antibody（552093） | PerCP-Cy™5.5 | BD Pharmingen |
| anti-Mouse CD11c Antibody（563735） | BV786 Hamster | BD Pharmingen |
| anti-Mouse I-A/I-E M5/114.15.2（557000） | PE Rat | BD Pharmingen |
| Fixable Viability Stain 510（564406） |  | BD Pharmingen |
