# Supplementary material for: Cryoablation synergizes with anti-PD-1 immunotherapy induces an effective abscopal effect in murine model of cervical cancer
Source: Transl Oncol. 2024 Nov 2;51:102175. doi: 10.1016/j.tranon.2024.102175 (PMC11565560; doi:10.1016/j.tranon.2024.102175)
Supplement: Supplementary file 4 [file mmc4.docx]

Table S2. The detailed list of gene sets for ssGSEA analysis

| **Signature name** | **Gene signature** |  |
| --- | --- | --- |
| T cell cytotoxicity | Cd3e,Cd3g,Cd4,Cd8a,Gzma,Gzmb,Gzmk,Ifng,Tbx21,Gzmc |  |
|  |  |  |
| T cell co-stimulation | Cd2,Cd226,Cd27,Cd28,Cd40lg,Icos,Slamf1,Tnfrsf18,Tnfrsf25 |  |
|  | Tnfrsf4,Tnfrsf8,Tnfrsf9,Tnfsf14 |  |
| Th1 cells | Ifng,Tbx21,Ctla4,Stat4,Cd38,Il12rb2,Lta,Csf2 |  |
